# The Relationship Between eHealth Literacy and Self-Efficacy Levels in Midwifery Students Receiving Distance Education During the COVID-19 Pandemic

**DOI:** 10.1097/jnr.0000000000000474

**Published:** 2022-03-02

**Authors:** Seda SÖGÜT, Eda CANGÖL, İlknur DOLU

**Affiliations:** 1 PhD, RN, Assistant Professor, Department of Midwifery, Faculty of Health Sciences, Çanakkale Onsekiz Mart University, Çanakkale, Turkey; 2 PhD, RN, Assistant Professor, Department of Midwifery, Faculty of Health Sciences, Bartin University, Bartin, Turkey.

**Keywords:** COVID-19, literacy, eHealth, midwifery, self-efficacy

## Abstract

**Background:**

During the ongoing COVID-19 pandemic period, most university courses in Turkey have been taught via distance education. Beyond knowledge of digital technologies, self-efficacy is known to affect the learning motivation and learning goals of students.

**Purpose:**

This study was conducted to determine the relationship between eHealth literacy and self-efficacy levels in midwifery students receiving distance education during the current COVID-19 pandemic. The research data were collected using a literature-based questionnaire developed by the researchers, the eHealth Literacy Scale for Adolescents, and the Online Technologies Self-Efficacy Scale. The data were analyzed using IBM SPSS Statistics 25.0, with values of *p* < .05 considered to be significant.

**Methods:**

This cross-sectional study was carried out during the COVID-19 pandemic on a sample of 578 female midwifery students. Snowball sampling method was used for data collection using an online questionnaire.

**Results:**

On the basis of the results, eHealth literacy and self-efficacy levels were relatively low in students who were 20 years old or below, who were in their first year, who were from low-income families, who spent less than an hour a day on the Internet, who had a low level of satisfaction with distance education, and who wanted to continue taking theoretical courses via distance education. In addition, self-efficacy related to online technologies was shown to be relatively low in students who found Internet services to be expensive, who had Internet connection problems, and who preferred asynchronous courses. Furthermore, a significant relationship was found between eHealth literacy and self-efficacy levels for online education.

**Conclusions/Implications for Practice:**

In the context of distance education, integrating the teaching/promotion of self-efficacy with regard to eHealth literacy and online technologies into midwifery education curriculum should better enable midwives to increase the quality of healthcare they provide and improve patient safety.

## Introduction

The issue of health literacy, although a relatively new concept in health promotion that came into general acceptance only in the late 20th century, is today an important concern in public health. The concept of health literacy encompasses a wide range of social and environmental interventions that are designed to benefit and protect health and quality of life by addressing and preventing the root causes of disease (World Health Organization, 2020). Health literacy is defined as having knowledge, motivation, and competence necessary to access, understand, evaluate, and apply information in daily life to make decisions about health services, disease prevention, and health promotion and to protect and enhance quality of life (Duong et al., 2018). The concept of eHealth literacy was elucidated in Norman and Skinner (2006) and defined as the ability to define, understand, and apply the information gained as a result of research on the Internet to provide solutions to health problems (Norman & Skinner, 2006). eHealth literacy plays an important role in increasing the capacity of students who are trained in the field of health to provide, interpret, and understand the basic health information and services necessary to make correct decisions related to patient health (Sharma et al., 2019).

Access to and selection of correct health information is very important for healthcare professionals (S. Kim & Jeon, 2020). Choosing correct health information for healthcare professionals is a basic skill gained in the undergraduate education process that further develops after graduation in line with one's area of specialization. Learning success relies heavily on a student's motivation, attitude, and self-efficacy, and technological developments in recent years have affected student learning abilities (S. Kim & Jeon, 2020; Sharma et al., 2019).

Digital technologies have become an integral part of modern education and changed the methods by which students learn (Tang & Chaw, 2016). Distance education, which has become widespread worldwide with the advance of digital technologies, is a method of education that provides easy and convenient access to learning opportunities through online learning resources such as online libraries and digital media (Prior et al., 2016). The effectiveness of distance education also relates closely to the digital technology self-efficacy of students (S. Kim & Jeon, 2020; Prior et al., 2016). In addition, the widespread use of online resources to access information about diseases and treatment by individuals in the community (Parnell et al., 2019), factors affecting health literacy level in terms of literature selection (H. Kim & Xie, 2017; Manganello et al., 2017), and undergraduate students' competence in health literacy (Sharma et al., 2019) should all be considered when designing online education content.

Midwives are health professionals who play an important role in the health, education, and care of patients. Midwifery students attend both theoretical and practical courses, including laboratory and clinical practice, during their undergraduate education that prepare them well for their professional responsibilities. The proliferation of the Internet and mobile phones has provided a very powerful platform for online learning that has been used to transfer knowledge, skills, and continuity of education in undergraduate midwifery education settings (Briones, 2015; J. H. Kim & Park, 2019). Some compulsory courses in Turkey such as foreign languages, Turkish literature, and basic information technology are already provided online to all undergraduate students, including midwifery students. Thus, midwifery students in Turkey have an extant familiarity with the online education experience (Kaçan & Gelen, 2020). During the COVID-19 pandemic, midwifery education and training in Turkey was reformatted completely to be delivered online. Midwifery students were quickly forced to adapt to this new education model for both practicum and theoretical courses. Therefore, the aim of this study was to determine the relationship between eHealth literacy and the learning self-efficacy levels of midwifery students in the context of distance education provided during the COVID-19 pandemic.

## Methods

### Design, Data Collection, and Sample

Female midwifery students currently enrolled in a midwifery undergraduate program in Turkey were recruited as participants. Male midwifery students were not included because of the small number of male students enrolled in these programs in Turkey (Higher Education Institution, 2019). This study was conducted between June 5, 2020, and July 1, 2020 (i.e., during the 2019–2020 spring term). Snowball sampling was used for data collection. One midwifery student began to share an online questionnaire voluntarily through her social media accounts with the request that the questionnaire be reshared. In total, 578 midwifery students enrolled as participants.

The inclusion criteria for this study were as follows: (a) being a female midwifery student, (b) volunteered to participate, and (c) at least 70% of courses attended are online. The exclusion criterion was being unwilling to participate.

The national core education program for undergraduate midwifery was delivered via distance education in the Spring Term 2019–2020 (Higher Education Institution, 2016) because of the ongoing COVID-19 pandemic. Courses were designed as synchronous lessons of, at most 20 minutes, using a videoconferencing application run through the universities' information management systems. All of the synchronous lessons were recorded and shared with students through these systems. The information management systems of universities enable lecturers to send instant or time-interval online examinations, case studies, and other materials, and to give feedback for each, and enable students to submit their video-recorded activities and written examinations.

### Measures

To collect research data, an online questionnaire prepared by the researchers using the literature, the eHealth Literacy Scale (eHEALS) for Adolescents, and the Online Technologies Self-Efficacy Scale (OTSES). All questions were mandatory.

### Questionnaire

The questionnaire consists of two parts. Respondent demographic information, including age, grade, place of residence, family structure, and income status, was collected in the first part. Questions on distance education, time spent on the Internet, Internet connection problems, satisfaction with distance education, the effect of distance education on student learning, whether theoretical and applied courses should continue to be given by distance education, and views on distance education were collected in the second part.

### eHealth Literacy Scale for Adolescents

eHEALS was developed by Norman and Skinner in 2006 to assess traditional literacy, health literacy, obtaining information, scientific research, media literacy, and computer literacy. This scale consists of eight items that assess the perception of respondents regarding using the Internet in health-related issues. Scale items are arranged using a 5-point Likert-type scale, with 1 = *strongly disagree*, 2 = *disagree*, 3 = *indecisive*, 4 = *agree*, and 5 = *strongly agree*. The range of total possible scores is 8–40, with higher scores indicating a higher eHealth literacy level. The Cronbach's alpha for eHEALS was found to be .88 in the first development study and .78 in a 2014 evaluation of the validity and reliability of the Turkish version of eHEALS in adolescents (Coşkun & Bebiş, 2015). The Cronbach's alpha value of the scale in this study was found to be .94.

### Online Technologies Self-Efficacy Scale

The validity and reliability of the Turkish version of the OTSES, originally developed by Miltiadou and Yu (2000), was performed by Horzum and Cakir (2009) with a Cronbach's alpha value of .94. The “Internet Competency” subscale covers four subscales consisting of nine items, the “Synchronous Interaction” subscale includes four items, the “Asynchronous Interaction 1” subscale includes nine items, and the “Asynchronous Interaction 2” subscale includes seven items. The range of total possible scores for the OTSES is 29–145, with higher scores indicating higher levels of self-efficacy perception. In this study, the Cronbach's alpha value was found to be .98 for the total of the scale and ranged from .92 to .97 for the subscales.

### Ethical Considerations

This study was approved by the Scientific Research Council of Bartin University (reference number: 2020-SBB-0108/June 3, 2020). All of the recruited students were informed of the objective of the study and invited to participate in the study via an email message. To ensure the confidentiality of participant information, no identification information was collected in the online questionnaire.

### Data Analysis

Descriptive statistics of frequencies, percentages, means, and standard deviations were calculated. Frequency, percentage, mean, and standard deviation were used to report the demographic and distance education characteristics. Independent samples *t* test, analysis of variance, and Pearson correlation coefficient were used to analyze normally distributed data, and Mann–Whitney *U* and Kruskal–Wallis tests were used to analyze nonnormally distributed data. All data were analyzed using IBM SPSS Statistics 25.0 (IBM Inc., Armonk, NY, USA), and level of significance was set to *p* < .05.

## Results

The average age of the 578 participants was 21 (*SD* = 1.83) years. One third (34.4%) were first-year students and lived in a big city, 82.5% came from a nuclear family, and 68.3% defined their family's income level as “median” (Table 1).

**Table 1.** Differences in eHealth Literacy and Online Technologies Self-Efficacy, by Demographic Characteristics (*N* = 578)

Category	*n*	%	eHEALS		OTSES									
					Internet Competencies Subscale		Synchronous Interaction Subscale		Asynchronous Interaction I Subscale		Asynchronous Interaction II Subscale		Total Score	
			Mean	*SD*	Mean	*SD*	Mean	*SD*	Mean	*SD*	Mean	*SD*	Mean	*SD*
Age (years; mean and *SD*)	21.10	1.83												
≤ 20	239	41.3	27.41	6.91	27.10	6.12	11.40	2.82	26.50	6.15	19.01	4.69	84.02	6.92
≥ 21	339	58.7	29.17	7.21	28.01	6.76	12.11	3.15	28.29	6.92	20.14	5.61	88.55	7.21
*t* and *p*			−2.943	.003	−1.640	.101	−2.787	.005	−3.218	.001	−2.546	.009	−2.732	.006
Program year														
① First year	199	34.4	27.43	6.62	27.39	6.01	11.50	2.84	26.79	5.88	19.12	4.70	84.80	17.41
② Second year	146	25.3	28.08	7.65	26.89	6.78	11.42	3.13	26.63	7.19	19.05	5.44	83.99	21.03
③ Third year	123	21.3	29.67	6.94	28.22	6.92	12.19	3.10	28.67	7.06	20.45	5.57	89.52	20.77
④ Fourth year	110	19.0	29.36	7.29	28.40	6.53	12.50	3.05	28.89	6.52	20.65	5.52	90.45	20.15
*F* and *p*			3.326	.019	1.568	.196	4.054	.007	4.496	.004	3.624	.013	3.728	.011
Post hoc, LSD			① < ③; ① < ④				① < ③; ① < ④; ② < ③; ② < ③		① < ③; ① < ④; ② < ③; ② < ④		① < ③; ① < ④; ② < ③; ② < ④		① < ③; ① < ④; ② < ③; ② < ④	
Residential area														
① Village	94	16.3	28.01	6.74	25.77	6.13	10.91	2.63	26.27	6.09	19.05	4.73	82.00	17.19
② District	155	26.8	29.42	6.36	28.48	5.86	12.14	2.82	28.32	5.88	19.96	5.02	88.90	17.84
③ Province	146	25.3	27.86	7.70	27.40	6.79	11.95	3.12	27.44	7.03	20.18	5.35	86.97	20.88
④ Metropol	183	31.7	28.31	7.44	28.06	6.85	11.91	3.27	27.64	7.21	19.35	5.66	86.96	21.33
*F* and *p*			1.438	.231	3.818	.010	3.533	.015	1.892	.130	1.263	.286	2.449	.063
Post hoc, LSD					① < ②; ① < ③; ① < ④		① < ②; ① < ③; ① < ④							
Family type														
Nuclear family	477	82.5	28.38	7.04	27.69	6.43	11.79	3.04	27.48	6.62	19.61	5.23	86.58	19.62
Single parent– extended family	101	17.5	28.75	7.58	27.36	6.94	11.94	3.03	27.88	6.88	19.96	5.49	87.14	20.51
*t* and *p*			22321 ^a^	.245	0.470	.639	−0.445	.656	−0.549	.583	0.599	.550	−0.259	.796
Income level														
① Low	74	12.8	25.32	9.02	25.96	8.09	11.24	3.44	26.28	7.86	18.24	6.00	81.73	23.69
② Median	395	68.3	28.94	6.71	27.54	6.28	11.85	2.91	27.57	6.49	19.70	5.06	86.65	18.92
③ High	109	18.9	28.74	6.70	29.12	5.89	12.09	3.15	28.35	6.33	20.56	5.38	90.12	19.30
*F* and *p*			8.353	< .001	5.402	.005	1.798	.167	2.127	.120	4.310	.014	4.014	.019
Post hoc, LSD			① < ②; ① < ③		① < ③; ② < ③						① < ②; ① < ③		① < ②; ① < ③	

***Note.*** Age range: 18–33 years. eHEALS = eHealth Literacy Scale; OTSES = Online Technologies Self-Efficacy Scale; LSD = least significant difference.

^a^ Mann-Whitney *U* Test.

### Differences in eHealth Literacy

The average eHEALS total score was 28.44 (*SD* = 7.13; Table 2). A statistically significant difference was found between eHEALS score and several demographic factors and perspectives on distance education. Participants who were < 20 years old (*t* = 2.943, *p* = .003), were in their first year (*F* = 3.326, *p* = .019), and identified their family income as “low” (*F* = 8.353, *p* < .001) earned relatively lower eHEALS scores (Table 1). Similarly, those who spent less than an hour a day on the Internet (*F* = 5.233, *p* < .001), had low satisfaction with distance education (*KW* = 18.163, *p* < .001), and wanted to continue theoretical courses through distance education (*t* = 2.314, *p* = .021) earned relatively lower eHEALS scores (Table 3).

**Table 2.** Correlations Between eHealth Literacy and Online Technologies Self-Efficacy (*N* = 578)

Variable	*M*	*SD*	1	2	2a	2b	2c	2d
1. eHealth Literacy Scale	28.44	7.13	1					
2. Online Technologies Self-Efficacy Scale	86.68	19.76	.541** [0.17, 0.22]	1				
2a. Internet Competency	27.63	6.52	.533** [0.51, 0.66]	.928** [2.72, 2.91]	1			
2b. Synchronous Interaction	11.82	3.04	.472** [0.94, 1.28]	.907** [5.68, 6.13]	.826** [1.67, 1.87]	1		
2c. Asynchronous Interaction 1	27.55	6.67	.507** [0.47, 0.62]	.943** [2.72, 2.88]	.833** [0.77, 0.86]	.803** [0.34, 0.39]	1	
2d. Asynchronous Interaction 2	19.67	5.28	.458** [0.52, 0.72]	.886** [3.18, 3.46]	.714** [0.81, 0.95]	.714** [0.42, 0.48]	.780** [0.92, 1.05]	1

**Correlation is significant at the .01 level (two-tailed).

**Table 3.** Differences in eHealth Literacy and Online Technologies Self-Efficacy, by Participant Perceptions of Distance Education (*N* = 578)

Category	*n*	%	eHEALS		OTSES									
			Mean	*SD*	Internet Competencies Subscale		Synchronous Interaction Subscale		Asynchronous Interaction I Subscale		Asynchronous Interaction II Subscale		Total Score	
					Mean	*SD*	Mean	*SD*	Mean	*SD*	Mean	*SD*	Mean	*SD*
Daily time spent on the Internet														
① 1 hour and less	30	5.2	23.63	8.74	23.63	9.52	10.40	3.84	23.60	8.04	17.27	5.72	74.90	25.76
② 2–4 hours	247	42.7	28.70	6.40	27.69	5.86	11.67	2.85	27.39	6.18	19.60	4.92	86.35	18.05
③ 5–7 hours	199	34.4	28.42	7.48	27.57	6.96	11.85	3.16	27.36	7.07	19.59	5.53	86.37	21.16
④ 8 hours and more	102	17.6	29.27	7.18	28.79	5.64	12.54	2.81	29.48	5.97	20.73	5.29	91.54	17.42
*F* and *p*			5.233	.001	4.962	.002	4.386	.005	6.658	< .001	3.510	.015	5.789	.001
Post hoc, LSD			① < ②; ① < ③; ① < ④		① < ②; ① < ③; ① < ④		① < ②; ① < ③; ① < ④; ② < ④; ③ < ④		① < ②; ① < ③; ① < ④; ② < ④; ③ < ④		① < ②; ① < ③; ① < ④		① < ②; ① < ③; ① < ④; ② < ④; ③ < ④	
Problems with Internet connection														
① None	298	51.6	29.02	7.06	28.75	6.13	12.18	3.02	28.38	6.34	20.10	5.30	89.41	19.02
② Interruption	143	24.7	28.38	6.98	27.39	6.49	11.80	2.91	27.34	6.92	19.79	5.23	86.31	19.76
③ High price	101	17.5	26.95	7.28	24.68	6.24	10.61	2.86	25.50	6.52	17.88	5.03	78.68	19.12
④ High price and interruption	36	6.2	28.11	7.57	27.61	7.96	12.31	3.38	27.31	7.55	20.72	5.08	87.94	22.11
*F* and *p*			2.159	.092	10.378	< .001	7.234	< .001	4.855	.002	5.142	.002	7.730	< .001
Post hoc, LSD			① > ③		① > ②; ① > ③; ③ < ④		① > ③; ② > ③; ③ < ④		① > ③; ② > ③		① > ③; ② > ③; ③ < ④		① > ③; ② > ③; ③ < ④	
Satisfaction with distance education														
① Very low	147	25.4	26.85	7.37	25.99	7.38	10.96	3.25	26.05	7.35	18.73	5.50	81.73	21.45
② Low	240	41.5	27.96	6.65	27.56	6.08	11.84	2.87	27.59	6.49	19.53	5.17	86.54	18.96
③ Moderate–high	191	33.0	30.28	7.17	28.97	6.05	12.46	2.91	28.65	6.12	20.58	5.11	90.65	18.59
*F* and *p*			18.163*	< .001	8.917	< .001	13.782*	.001	6.582	.002	5.283	.005	13.024*	.001
Post hoc, LSD			② < ③		① < ②; ① < ③; ② < ③		① < ②		① < ②; ① < ③		① < ③; ② < ③		① < ②; ② < ③	
Impact of distance education on midwifery students' learning process														
Very low	231	40.0	27.83	6.82	26.93	6.90	11.50	3.10	26.94	6.98	19.09	5.39	84.46	20.2
Low	239	41.3	28.44	7.09	28.07	6.27	12.04	2.99	28.01	6.49	20.06	5.21	88.18	19.47
Moderate–high	108	18.7	29.75	7.75	28.18	6.11	12.00	2.96	27.85	6.30	20.07	5.10	88.10	19.09
*F* and *p*			2.219	.085	4.459*	.216	2.103	.123	3.637**	.303	2.367	.095	6.342**	.096
Preference for attending theoretical course through distance education														
No	379	65.6	27.95	7.15	27.10	6.83	11.60	3.09	27.11	6.96	19.46	5.41	85.27	20.50
Yes	199	34.4	29.39	7.02	28.64	5.75	12.24	2.89	28.38	5.99	20.09	5.00	89.35	18.03
*t* and *p*			−2.314	.021	−2.716	.007	−2.437	.015	−2.1814	.030	−1.357	.175	−2.368	.018
Preference for attending practical course through distance education														
No	498	86.2	28.30	7.00	27.49	6.50	11.74	3.01	27.49	6.63	19.64	5.23	86.36	19.62
Yes	80	13.8	29.31	7.89	28.53	6.56	12.33	3.16	27.93	6.91	19.89	5.56	88.66	20.64
*t* and *p*			−1.175	.241	−1.320	.187	−1.611	.108	−.542	.588	−.388	.698	−.968	.333
Type of distance education														
Live	330	57.1	28.80	7.45	28.09	6.85	12.21	3.02	28.19	6.92	20.50	5.48	88.99	20.77
Video	204	35.3	27.85	6.63	26.67	5.94	11.13	3.02	26.32	6.16	18.30	4.70	82.43	17.63
Both	44	7.6	28.55	6.88	28.64	6.12	12.07	2.77	28.45	6.29	19.84	5.03	89.00	18.42
*F* and *p*			1.121	.327	3.601	.028	8.298	< .001	5.453	.005	11.337	< .001	7.436	.001
Post hoc, LSD					① > ②		① > ②		① > ②		① > ②		① > ②; ② < ③	

***Note.*** eHEALS = eHealth Literacy Scale; OTSES = Online Technologies Self-Efficacy Scale; LSD = least significant difference.

*p < .05. **p < .01.

### Differences in Online Technologies Self-Efficacy

The average OTSES total score was 86.68 (*SD* = 19.76), whereas the average score was 27.63 (*SD* = 6.52) for the “Internet Competency” subscale, 11.82 (*SD* = 3.04) for the “Synchronous Interaction” subscale, 27.55 (*SD* = 6.67) for the “Asynchronous Interaction 1” subscale, and 19.67 (*SD* = 5.28) for the “Asynchronous Interaction 2” subscale (Table 2). A statistically significant difference was found between total OTSES scores and subscale scores and several demographic factors and distance education perspectives. Participants who were < 20 years old, were in their first or second year (*t* = 2.732, *p* = .006), and identified their family income as “low” (*F* = 2.943, *p* = .003) earned relatively low OTSES total scores (Table 1). In addition, those who spent an hour or less on the Internet earned relatively lower OTSES total scores than their peers who spent more time on the Internet. In addition, those who spent 2–7 hours on the Internet earned a lower OTSES total score than their peers who spent ≥ 8 hours on the Internet (*F* = 2.943, *p* = .003). The participants who perceived Internet services as expensive earned OTSES scores that were relatively lower than those who experienced disconnection, who perceived high price and experienced Internet disconnection problems, and who experienced no problems (*F* = 7.730, *p* < .001).

OTSES scores (*KW* = 13.024, *p* = .001) were found to decrease as dissatisfaction with distance education decreased, and satisfaction was found to be lower in those participants who expressed a preference for not attending theoretical courses via distance education (*t* = 2.368, *p* = .018). In addition, the OTSES scores (*F* = 7.436, *p* = .001) of those who preferred asynchronous courses were found to be lower than those who preferred synchronous courses or both asynchronous and synchronous courses (Table 3).

### Relationship Between the eHealth Literacy and Online Technologies Self-Efficacy Scales

eHEALS was found to be statistically significantly related to the OTSES (*r* = .541, *p* < .01). In addition, significant relationships were found between eHEALS and the “Internet Competency” (*r* = .533, *p* < .01), “Synchronous Interaction” (*r* = .472, *p* < .01), “Asynchronous Interaction 1” (*r* = .507, *p* < .01), and “Asynchronous Interaction 2” (*r* = .458, *p* < .01) subscales of the OTSES (Table 2).

## Discussion

This study was designed to determine the relationship between midwifery students' self-efficacy with regard to online technologies and eHealth literacy levels in the distance education process during the COVID-19 pandemic. Similar studies on nursing students indicate that more than half of students have high levels of eHealth literacy (S. Kim & Jeon, 2020; Tubaishat & Habiballah, 2016). In this study, eHEALS was calculated as 28.44, which is a level similar to that found in a study of nursing students by Rathnayake and Senevirathna (2019).

In Knapp et al. (2011), the eHealth literacy level of individuals with low education and family income levels was found to be low. In contrast to this study, no significant difference was found in similar studies on age and eHealth literacy (Rathnayake & Senevirathna, 2019; Tubaishat & Habiballah, 2016). Significant differences were found in this study between sociodemographic factors such as age, income level, and educational level and the student's capacity to interpret and understand the basic health information and services required to make correct health decisions. The low eHEALS scores among the first-year students aged ≤ 20 years may be associated with the newness of their midwifery education and of their understanding/interpretation of health information.

Frequency of Internet use and ability to use the Internet are important factors in improving health literacy. In this study, those participants who spent less than an hour a day on the Internet, expressed low satisfaction with distance education, and wanted to continue theoretical courses through distance education earned lower eHEALS scores. Studies show that level of eHealth literacy increases as Internet-usage skills increase (J. H. Kim & Park, 2019; S. Kim & Jeon, 2020; McCutcheon et al., 2018; Zheng et al., 2018). The results of this study were similar to those of other studies. Therefore, it is very important to consider whether education is properly provided to improve eHealth literacy ability and develop eHealth literacy skills from the first year in midwifery students.

Distance education also affects the self-efficacy of students in terms of increasing their learning outcomes and capacity for learning. Self-efficacy is important for distance education in terms of course completion, peer and learning interactions, and teacher interaction (Yavuzalp & Bahcivan, 2020; Zimmerman & Kulikowich, 2016). In this study, the average total OTSES score was found to be moderate, which is consistent with the findings of Jan (2015) and Lee (2015). In this study, the total OTSES scores of those participants who were < 20 years old, were in their first and second years, and reported their family income level as “low” were lower than those of their peers. In Jan, self-efficacy was found to be lower in men than in women, and no significant difference was found across age groups. The results of this study reveal the importance of developing self-efficacy skills from the first (freshman) year in midwifery students via online education.

In this study, similar to Zimmerman and Kulikowich (2016), a positive relationship was found between the previous experiences and the online self-efficacy levels of participants receiving distance education. In this study, the total OTSES scores of those who spent an hour or less on the Internet were found to be lower. Similarly, those who found Internet services expensive had lower OTSES scores compared with those who experienced disconnection, who found Internet services to be expensive and experienced disconnection, and who experienced no problems. The findings show that OTSES scores decreased as distance education satisfaction decreased. However, low OTSES scores were also found in those who did not want to take theoretical courses via distance education. Previous academic studies also echo these findings (Prior et al., 2016; Zimmerman & Kulikowich, 2016). It is anticipated that previous experience will positively influence the efficiency of students who are willing to be part of distance education.

Higher online self-efficacy is positively associated with satisfaction with distance education (Jan, 2015; Prior et al., 2016). In distance education, eHealth literacy is positively related to self-efficacy (Prior et al., 2016). In this study, eHEALS was found to be statistically significantly related to the OTSES, which is consistent with the results of other relevant studies (Jan, 2015; S. Kim & Jeon, 2020). The results of this study support that self-efficacy toward online technologies increases satisfaction in distance education as well as the success of education.

### Limitations of the Study

Online platforms regularly used by female midwifery students were used to collect the data for this study. Therefore, the findings may not be generalizable to all midwifery students. In addition, students' opinions may be influenced by different applications used by universities, and it is considered that the use of distance education technologies for midwifery courses in all universities at the same time because of the pandemic is a factor that reduces this risk. In addition, as only female midwifery students were included in the study, no gender comparison could be made. The average age of the participants in the study was 21 years. As younger generations are better prepared to use digital technologies well, it is important to examine the self-efficacy of midwives already working in the field and the effects of health-related decisions on healthcare decisions.

### Conclusions

This study was conducted on midwifery students to assess the relationship between self-efficacy toward online technologies and eHealth literacy levels in distance education during the COVID-19 pandemic. On the basis of the results, eHealth literacy and self-efficacy levels were low in the participants who were 20 years old or below, were in their first year of the midwifery program, were from a low-income family, spent less than an hour a day on the Internet, had a low level of satisfaction with distance education, and wanted to continue taking theoretical courses via distance education. This study also found a low level of self-efficacy for online technologies in those students who were 20 years old or below, were in their first or second year, were from a low-income family, perceived Internet services as expensive, experienced Internet connection problems, had a low level of satisfaction with distance education, did not want to continue theoretical courses via distance education, and preferred asynchronous lessons. In addition, a relationship was identified in the participants between eHealth literacy and self-efficacy for online education.

The findings obtained from this study provide basic data for educational managers and educators to better support students with appropriate training programs to increase eHealth literacy and self-efficacy. In distance education, integrating the concept of self-efficacy for eHealth literacy and online technologies into the midwifery education curriculum is expected to enable midwives to make decisions that will enhance the quality of the healthcare they provide and the safety of their patients.
